# Eletrocardiograma de Jogadores de Futebol de Elite Brasileiros: Preenchendo uma Lacuna

**DOI:** 10.36660/abc.20230090

**Published:** 2023-04-18

**Authors:** Ricardo Stein, Filipe Ferrari, Anderson Donelli da Silveira

**Affiliations:** 1 Programa de Pós-Graduação em Cardiologia e Ciências Cardiovasculares Universidade Federal do Rio Grande do Sul Porto Alegre RS Brasil Programa de Pós-Graduação em Cardiologia e Ciências Cardiovasculares, Universidade Federal do Rio Grande do Sul, Porto Alegre, RS – Brasil; 2 Grupo de Pesquisa em Cardiologia do Exercício Hospital de Clínicas de Porto Alegre Porto Alegre RS Brasil Grupo de Pesquisa em Cardiologia do Exercício do Hospital de Clínicas de Porto Alegre (CardioEx-HCPA), Porto Alegre, RS – Brasil; 3 Departamento de Clínica Médica Universidade Federal do Rio Grande do Sul Porto Alegre RS Brasil Departamento de Clínica Médica da Universidade Federal do Rio Grande do Sul, Porto Alegre, RS – Brasil

**Keywords:** Atletas, Exercício, Morte Súbita Cardíaca, Parada Cardíaca, Eletrocardiografia/métodos, Programa de Rastreamento, Futebol, Origem Étnica e Saúde

## Introdução

A morte súbita de um atleta aparentemente saudável é uma tragédia, e a real incidência de morte súbita cardíaca (MSC) em uma população de atletas é controversa. Embora estimativas de estudos conduzidos nos Estados Unidos (EUA) e na Europa sejam limitadas por várias questões metodológicas, sabe-se que o esforço físico em atividades esportivas e treinos aumenta em 2,5 a 4,5 o risco de parada cardíaca e MSC em relação ao observado em não atletas e atletas recreativos.^1 - 3^ Por outro lado, um achado consistente nos estudos é o fato de que atletas do sexo masculino têm uma incidência de três a cinco vezes maior de MSC que atletas do sexo feminino.^4^

Ao longo das últimas décadas, muito tem se discutido acerca do papel do eletrocardiograma de repouso de 12 derivações (ECG) na prevenção de MSC em atletas jovens. Estudos conduzidos em diferentes partes do mundo foram publicados, e consensos de especialistas foram desenvolvidos a fim de padronizar a realização desse tipo peculiar de ECG.^4 - 6^

Segundo o critério internacional de interpretação de ECG em atletas,^4^ um documento que representou um consenso internacional de experts para a interpretação de ECG em atletas, a inclusão do exame na avaliação pré-participação foi eficaz na detecção das doenças estruturais mais comuns, principalmente cardiomiopatia hipertrófica e cardiomiopatia arritmogênica do ventrículo direito e/ou esquerdo, além de algumas canalopatias.

Vale mencionar que, em uma coorte de jogadores de futebol adolescentes no Reino Unido, o número de atletas com ECG anormal foi reduzido em 57% ao se aplicar o critério internacional (1,8%) no lugar do critério de Seattle (4,3%).^7^ Do ponto de vista do Brasil, parece óbvio que cardiologistas e médicos do esporte devam compreender as nuances do ECG do atleta, um exame aparentemente simples, mas com inúmeras particularidades. Além disso, considerando que o Brasil é um país com milhares de jogadores de futebol, seria importante conhecer o padrão eletrocardiográfico de jovens jogadores, uma vez que as informações disponíveis, tanto clínicas como para fins de pesquisa, são provenientes de coortes internacionais que podem não representar adequadamente nossa população.

## Achados eletrocardiográficos em jogadores de futebol de elite Brasileiros

O futebol é um esporte muito popular no Brasil, e a relação entre o esporte e o país é única, e reconhecida mundialmente. Contudo, recomendações específicas sobre o ECG para atletas brasileiros nunca foram publicadas. Ainda, há falta de dados consistentes obtidos de uma grande coorte de jogadores profissionais de futebol em nosso país.

A necessidade de informações refinadas, em larga escala, sobre o ECG de atletas brasileiros merece nossa atenção e deve ser prioridade por várias razões. Primeiro, dados de coortes internacionais, com históricos e prevalências de achados diferentes (por exemplo: o padrão de onda T em pessoas africanas e afro-caribenhas), são rotineiramente usados para a avaliações de atletas brasileiros. Segundo, o comércio de escravos no passado distante, e posterior imigração de europeus a regiões específicas do Brasil, resultou em uma população composta por grupos étnicos intensamente miscigenados, com contrastes regionais marcantes. Por isso, a prevalência dos achados eletrocardiográficos dos indivíduos pode se diferenciar de acordo com seu local de nascimento.

Assim, o que explicaria a falta de dados de jogadores de futebol brasileiros apesar de sua importância clínica? Acreditamos que isso se deve a fatores como a falta de esforços coordenados, a ausência de cardiologistas e a falta de coletas de dados em clubes profissionais. Por sinal, construir um banco de dados com atletas de todas as regiões do país para se obter uma amostra realmente representativa é desafiador, porém necessário.

A questão da etnia é particularmente importante, uma vez que ela surgiu nas últimas décadas como um dos principais determinantes de alterações e adaptações cardiovasculares em atletas. Um dos avanços nessa área foi o reconhecimento do padrão “africano/afro-caribenho”^8^ como uma variante normal em atletas dessas etnias (ou seja, inversão da onda T nas derivações V1-V4, precedida por elevação do ponto J e elevação convexa do segmento ST).^9^ Após extensa investigação, não foram encontrados sinais de doença cardíaca nesses atletas, sendo esse padrão adicionado nos critérios internacionais para interpretação do ECG em atletas.^4^ Ainda, já foi bem documentado que atletas negros apresentam maior prevalência de hipertrofia ventricular esquerda, repolarização precoce, e inversão da onda T em comparação a atletas brancos.^4^

No Brasil, há uma prevalência significativa de jogadores de futebol pardos (mestiços), e tal mistura de raças não foi contemplada nas diretrizes sobre ECG de atletas. As recomendações são predominantemente para atletas brancos e negros, e essa classificação binária é inadequada e sujeita a erro em muitos aspectos na avaliação de jogadores de futebol brasileiros. De fato, dados oriundos de atletas pardos são escassos. Malhotra et al.,^7^verificaram que atletas pardos apresentaram uma maior prevalência de inversão da onda T nas derivações inferiores, mas ainda há muito o que aprender. Por exemplo, qual a prevalência e a importância prognóstica do padrão “africano/afro-caribenho” nos atletas de etnia parda? Devemos lembrar que esses atletas possivelmente tenham alguma ancestralidade negra.

**Figura 1:**
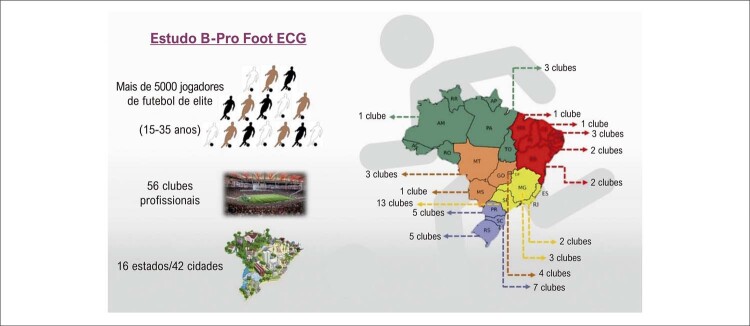
– Projeto “B-Pro Foot ECG Study”.

Por outro lado, nosso grupo (CardioEx-HCPA) está analisando quais atletas de etnia parda apresentam essa variante e quais os achados ecocardiográficos correspondentes. Nossa hipótese é a de que respostas a essas perguntas possam guiar estratégias mais adequadas para a interpretação do ECG desses atletas. De fato, para superar a escassez de dados relacionados à cardiologia do esporte no Brasil, nosso grupo tem tentado esclarecer essas e outras questões. Por exemplo, estamos avaliando uma série de variáveis eletrocardiográficas de uma grande coorte de jogadores de futebol de elite brasileiros de etnia branca, parda, e negra, das cinco regiões do país (atualmente, mais de 5000 atletas com idade entre 15 e 35 anos, de 56 clubes profissionais localizados em 16 estados e 42 cidades), um projeto chamado “ *B-Pro Foot ECG Study* ” ( Figura 1 ).^9^ Demonstramos uma maior prevalência de achados eletrocardiográficos compatíveis com o “coração do atleta” em indivíduos negros em comparação a jogadores brancos ou pardos.^9^ Até o momento, nosso dados apresentaram uma prevalência de achados eletrocardiográficos anormais de aproximadamente 5% nos jogadores de futebol de elite brasileiros.

Outra questão relevante refere-se à porcentagem da variante “africana/afro-caribenha” nos jogadores brasileiros negros. Em um estudo transversal com 150 jogadores de futebol de elite de Gana, foi relatada uma prevalência de aproximadamente 19% desse padrão.^10^ Em um estudo conduzido por Papadakis et al.,^8^ essa variante foi detectada em cerca de 13% dos atletas negros de origem africana/afro-caribenha. Em nossa amostra de mais de 1.300 jogadores de futebol de elite negros, essa prevalência parece ser significativamente mais baixa. Essa informação relacionada à etnia pode ajudar na tomada de decisão clínica quando o ECG é usado como um método propedêutico.

Esperamos que nossa iniciativa seja bem sucedida e encoraje outros grupos de pesquisa de nosso país a produzir evidência de qualidade nessa área do conhecimento. Ainda, ajudar com informação robusta para se definir quais achados eletrocardiográficos são normais e anormais para os jogadores de futebol de elite do Brasil. Outro desafio será avaliar as características eletrocardiográficas de jogadoras de futebol feminino, uma vez que esse é um esporte de rápida ascensão no Brasil.

## Conclusões

O ECG do atleta tem sido estudado principalmente no hemisfério norte, especialmente nos Estados Unidos e alguns países europeus. No Brasil, não conhecemos estudos que fornecem informações sobre o padrão eletrocardiográfico de jogadores de futebol profissionais dos diferentes grupos étnicos que constituem nossa população. Conceitos de validade interna *versus* validade externa são importantes e, com isso em mente, decidimos investigar quais informações, obtidas de coortes internacionais, poderiam se correlacionar com jogadores de futebol brasileiros.

Em um primeiro e amplo estudo descritivo, nosso objetivo será preencher uma lacuna nessa área do conhecimento. Em seguida, objetivamos comparar dados de outros países com os nossos dados (com base em mais de 6.000 ECGs), além de estabelecer o que seria normal, em termos de ECG, para os jogadores de etnia branca, parda ou negra no país do rei Pelé.
