# Supplementary material for: Eletrocardiograma de Jogadores de Futebol de Elite Brasileiros: Preenchendo uma Lacuna
Source: Arq Bras Cardiol. 2023 Apr 18;120(4):e20230090. [Article in Portuguese] doi: 10.36660/abc.20230090 (PMC10263416; doi:10.36660/abc.20230090)
Supplement: Translation

# Translation: Electrocardiogram of Brazilian Elite Football Players: Filling a Gap

**Authors:** Ricardo Stein, Filipe Ferrari, Anderson Donelli da Silveira

**Affiliations:** 1 Brasil Programa de Pós-Graduação em Cardiologia e Ciências Cardiovasculares, Universidade Federal do Rio Grande do Sul, Porto Alegre, RS – Brasil; 2 Brasil Grupo de Pesquisa em Cardiologia do Exercício do Hospital de Clínicas de Porto Alegre (CardioEx-HCPA), Porto Alegre, RS – Brasil; 3 Brasil Departamento de Clínica Médica da Universidade Federal do Rio Grande do Sul, Porto Alegre, RS – Brasil

**Keywords:** Athletes, Exercise, Sudden Cardiac Death, Heart Arrest, Mass Sreening, Soccer, Ethnicity and Health

## Introduction

The sudden death of a young, apparently healthy athlete is a tragedy, and the true incidence of sudden cardiac death (SCD) among athletes is controversial. Although estimates from studies in the United States and Europe are limited by various methodological issues, it is known that physical exertion in sports activity and training poses a 2.5 to 4.5 times higher risk of sudden cardiac arrest and SCD in relation to non-athletes and recreational athletes.^1 - 3^On the other hand, a consistent finding across studies is that male athletes have a three to five times higher incidence of SCD than female athletes.^4^

Over the past few decades, much has been discussed about the role of the resting 12-lead electrocardiogram (ECG) in preventing SCD in young athletes. Studies carried out in different parts of the world have been published, and expert consensuses have been developed to standardize a very peculiar type of ECG.^4 - 6^

According to the International criteria for electrocardiographic interpretation in athletes,^4^which represents an international expert consensus on ECG interpretation in athletes, the inclusion of the ECG in the pre-participation screening effectively detects the most common structural diseases, mainly hypertrophic cardiomyopathy and arrhythmogenic cardiomyopathy in the right and/or left ventricle, as well as some channelopathies.

It is worth of mention that in a cohort of 11,168 adolescent football players from the United Kingdom, the number of athletes with an abnormal ECG was reduced by 57% when moving from the Seattle Criteria (4.3%) to the International Criteria (1.8%).^7^From the Brazilian perspective, it seems obvious that cardiologists and sports physicians should understand the nuances of the athlete’s ECG, an apparently simple exam, but with countless peculiarities. In addition, since Brazil is a country with thousands of football players, it would be important to know the electrocardiographic pattern of young football players, since all available information either for clinical or research purposes comes from international cohorts that may not represent adequately our population.

## Electrocardiographic findings in Brazilian elite football players

Football is very popular in Brazil, and the relationship between the sport and the country is unique and renowned worldwide. However, specific recommendations regarding Brazilian athletes’ ECGs have never been published. Also, there is a lack of consistent data from a large cohort of professional football players in the country.

The need for refined and large-scale information on this issue deserves our attention and should be a priority for several reasons. First, data from international cohorts are routinely used for the assessment of these athletes, with different backgrounds and prevalence of specific findings (e.g., African/Afro-Caribbean T-wave pattern). Secondly, because of the slave trade in the distant past and the later European immigration to specific regions in Brazil, the population is composed of highly mixed ethnic groups, with notable regional contrasts. As a result, the prevalence of ECG findings may vary according to the place of birth.

So, what could explain this lack of data from Brazilian football athletes despite their clinical relevance? We believe that factors such as poorly coordinated efforts, the absence of cardiologists and of a structured data collections at professional clubs can explain this. Constructing a database with athletes from all regions of the country to reach a truly representative sample is challenging but needed.

The issue of ethnicity is particularly salient since it has emerged in recent decades as one of the main determinants of cardiovascular changes and adaptations in athletes. One of the advances in this area was the recognition of the “African/Afro-Caribbean pattern”^8^as a normal variant in athletes with this origin (i.e., T-wave inversion in leads V1-V4 preceded by J-point elevation and convex ST-segment elevation).^9^After extensive investigation, no signs of cardiac disease were found in these athletes, and this pattern was added to the international criteria for electrocardiographic interpretation in athletes.^4^Furthermore, it is well documented that black athletes have a higher prevalence of left ventricular hypertrophy, early repolarization, and T-wave inversion compared to white athletes.^4^

In Brazil, there is a significant prevalence of mixed-race football players, which has not been addressed in the guidelines on the athlete’s ECG. The recommendations are mainly for white and black athletes, and such binary classification is inadequate and subject to error in many aspects in the evaluation of Brazilian football players. In fact, data from mixed-race athletes are still scarce. Malhotra et al.^7^verified that mixed-race athletes had a higher prevalence of T-wave inversion in lower leads than white athletes, but there is still much to learn. For example, what is the prevalence and prognostic significance of the “African/Afro-Caribbean pattern” in mixed-race athletes? We should remember that these individuals possibly have some black ancestry.

On the other hand, our group (CardioEx-HCPA) is analyzing which Brazilian mixed-race athletes present this variant and what are the corresponding echocardiographic findings. It is our hypothesis that the answers to such questions can guide more appropriate strategies for the interpretation of the ECG of these athletes. In fact, to overcome the scarcity of data related to sports cardiology in Brazil, our group has tried to clarify these and other issues. For example, we are evaluating a series of ECG variables from a large cohort of elite Brazilian football players of white, mixed-race, and black ethnicity, from all five geographic regions of Brazil (currently more than 5,000 athletes aged 15 to 35 years, from 56 professional clubs in 16 states and 42 cities) – a project called the “B-Pro Foot ECG Study” ( Figure 1 ).^9^We have shown a higher prevalence of electrocardiographic findings compatible with the “athlete’s heart” in blacks in comparison to white or mixed-race male footballers.^9^Brazilian Afro-descendant players also appear to be associated with a higher prevalence of T-wave inversion in the inferior and/or lateral leads compared to white or mixed-race players. So far, our data have shown a prevalence of abnormal ECG findings of approximately 5% in elite Brazilian football players.

**Figure 1:** – “B-Pro Foot ECG Study” Project.

Another relevant question is the percentage of the “African/Afro-Caribbean” variant in black Brazilian players. In a cross-sectional study with 150 Ghanaian elite football players, a prevalence of nearly 19% of this pattern was reported.^10^In a study conducted by Papadakis et al.,^8^this variant was detected in approximately 13% of black athletes of African/Afro-Caribbean origin. In our sample of more than 1,300 black elite Brazilian football players, however, this prevalence this prevalence appears to be significantly lower. This ethnicity-related information can help in clinical decision-making when the ECG is used as a propaedeutic method.

We hope that our initiative will be successful and encourage other research groups in our country to produce high-quality evidence in this area of knowledge. Our goal is to provide robust information that can help distinguish between normal and abnormal ECG readings for elite Brazilian football players. Another challenge will be to evaluate electrocardiographic characteristics of female football players, since female football is a fast-growing sport in Brazil.

## Conclusions

The athlete’s ECG has been mainly investigated in the northern hemisphere, especially in the United States and in some European countries. In Brazil, we are not aware of studies that provide information about the ECG pattern of professional football players from the different ethnic groups that make up our population. Concepts of external *versus* internal validity matter, and with that in mind we decided to investigate what information, obtained from international cohorts could be correlated to Brazilian football players.

In a large descriptive study, we aim to fill a gap in this area of knowledge. For further investigations, we aim to compare data from other countries with ours (based on more than 6,000 ECGs), in addition to establish what is normal regarding ECG for the white, mixed-race, or black football players in the country of the king Pelé.
